# Informing road traffic intervention choices in South Africa: the role of economic evaluations

**DOI:** 10.3402/gha.v9.30728

**Published:** 2016-07-06

**Authors:** Hadley K.H. Wesson, Nkuli Boikhutso, Adnan A. Hyder, Melanie Bertram, Karen J. Hofman

**Affiliations:** 1 Department of Surgery, Johns Hopkins School of Medicine, Baltimore, MD, USA; 2 School of Public Health, University of Witwatersrand, Johannesburg, South Africa; 3 International Injury Research Unit, Bloomberg School of Public Health, Johns Hopkins University, Baltimore, MD, USA; 4 School of Public Health, University of Witwatersrand, PRICELESS SA, MRC/Wits Rural Public Health and Health Transitions Research Unit, Johannesburg, South Africa

**Keywords:** economic evaluation, cost-effectiveness analysis, injury, accidents, South Africa, low- and middle-income countries

## Abstract

**Introduction:**

Given the burden of road traffic injuries (RTIs) in South Africa, economic evaluations of prevention interventions are necessary for informing and prioritising public health planning and policy with regard to road safety.

**Methods:**

In view of the dearth of RTI cost analysis, and in order to understand the extent to which RTI-related costs in South Africa compare with those in other low- and middle-income countries (LMICs), we reviewed published economic evaluations of RTI-related prevention in LMICs.

**Results:**

Thirteen articles were identified, including cost-of-illness and cost-effectiveness studies. Although RTI-related risk factors in South Africa are well described, costing studies are limited. There is minimal information, most of which is not recent, with nothing at all on societal costs. Cost-effective interventions for RTIs in LMICs include bicycle and motorcycle helmet enforcement, traffic enforcement, and the construction of speed bumps.

**Discussion:**

Policy recommendations from studies conducted in LMICs suggest a number of cost-effective interventions for consideration in South Africa. They include speed bumps for pedestrian safety, strategically positioned speed cameras, traffic enforcement such as the monitoring of seatbelt use, and breathalyzer interventions. However, interventions introduced in South Africa will need to be based either on South African cost-effectiveness data or on findings adapted from similar middle-income country settings.

## Introduction

The Decade of Action for Road Safety 2011–2020, which is now at the halfway mark, began with goals to improve road and vehicle safety and increase the legislation and enforcement of the use of helmets, seatbelts, and child restraints; drink driving laws; and speed limits (1). Globally, efforts are underway to study these interventions, not only in terms of road traffic injuries (RTIs) and related deaths, but also their costs and cost-effectiveness. This is particularly relevant to low- and middle-income countries (LMICs) with constrained resources.

In South Africa, RTIs are a leading cause of injury-related deaths, accounting for 27 deaths per 100,000 people compared to the global average of 10 deaths per 100,000 (2). South Africa's injury-related mortality rate is higher than the aggregate death rate for the World Health Organization (WHO) African Region and nearly twice the global average. In 2012, RTIs in South Africa accounted for USD10.5 billion of health services expenditure, or 3% of gross domestic product (GDP) (3).

South Africa's RTI risk factors are well described by Statistics South Africa, the Road Transport Management Corporation, and the National Injury Mortality Surveillance System. These include lack of pedestrian safety measures, alcohol misuse, aggressive driving, and limited seatbelt use (4–6). Sixty percent of fatal RTIs are due to the influence of alcohol (4). Speeding is a factor in 30–50% of road traffic crashes (7). Concurrently, seatbelt use in South Africa is estimated to be 50%, at best, for front seat occupants, and 8% for rear-seated passengers (2). Seatbelt use is proportionally lower in lower-income areas within South Africa (8).

In 2008, the National Road Traffic Act introduced a number of safety requirements to address the risk factors outlined in Table 1, (9). However, over the last 8 years, implementing these legislative initiatives has been limited (10). In the absence of enforced legislation and targeted interventions, the costs of RTIs in South Africa are mounting, comprising more than 1.5 times South Africa's GDP per capita (4). Not only is this expenditure high compared to other LMICs, but it approaches the 3.8% of GDP allocated to all government public health spending in South Africa (11).

**Table 1:** Road traffic injury (RTI) safety requirements introduced in South Africa's National Road Traffic Act, 2008

RTI safety requirement
Cyclists wear helmets.
Child restraints are enforced.
Child pedestrian reflective clothing is evaluated.
Roadside alcohol testing is instituted.
Seatbelts must be functional.
Minibus taxis must provide seatbelts for drivers and at least one passenger.

Source: South African Department of Transport, 2008.

The aim of this study is three-fold. First, describe sources of information and the full extent to which RTI-related costing data are available in South Africa. Second, describe the extent to which RTI-related costing data are available in other LMICs through a review of the literature. Third, use these findings to suggest potential cost-effective RTI prevention interventions for South Africa.

## RTI data collection systems in South Africa

South African RTI-related data are collected by two independent organizations: the National Injury Mortality Surveillance System (NIMSS) and National Department of Transportation (NDOT). In 2008, NIMSS collected data from 39 mortuaries in seven of South Africa's nine provinces (5). The data are biased towards urban areas because the data from the rural mortuaries are concentrated in only one province. The data do not include costs.

The NDOT is the main source of RTI-related data, having published three reports to date (12–14). The first report, published in 2000, classified RTIs as fatal, severe, or minor from 1998 data collected by the Road Accident Fund (RAF) (12). The RAF is a statutory body that provides compulsory insurance to South African road users. In 2002, the NDOT published its second report: a cost of RTI survey based on 363 household interviews (13). The third report, published in 2004, analysed data from the RAF (14). The 2000 and 2004 publications reported the exact number and distribution of RTI fatalities; an additional study described the national costs associated with RTIs by referencing the 2000 report as its primary data source (15). In an effort to avoid duplication, we report only findings from the 2000 report that used 1998 data, emphasizing that South African costing studies are based on data that is now nearly 20 years old.

## Costs of RTIs in South Africa

**Fig. 1:**
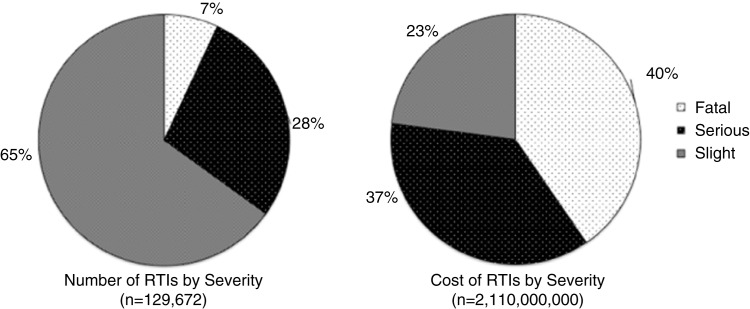
The total number and total costs of road traffic injuries (RTIs) in South Africa in 1998. Source: Department of Transport, South Africa (12).

**Fig. 2:**
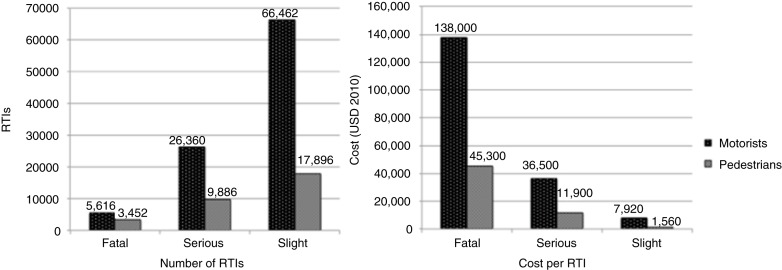
The total number and total costs of road traffic injuries (RTIs) in South Africa in 1998, by severity and status (costs reported in USD 2010). Source: Department of Transport, South Africa (12).

In 1998, there were 129,672 RTIs that cost more than USD 1.57 billion, or USD 2.1 billion, when converted to 2010 values, although the type of costs included in this estimate is not stated (12). Seven percent of these RTIs were fatal and accounted for 40% of the total costs; slight injuries accounted for 65% of RTIs but only 23% of costs (Fig. 1) (12). In contrast, pedestrian injuries accounted for 24% of all RTI-related injuries, but only 13% of total costs. Fatal and severe pedestrian injury costs were much lower than similar motorist expenditures (Fig. 2) (12). The NDOT did not define ‘serious’ and ‘slight’ injuries, limiting the ability to generalize findings to other studies.

In 2009, alcohol-related RTIs in South Africa resulted in USD 940.6 million in damage to motor vehicles (16). Only one study has looked at cost-effectiveness with regard to seatbelts and RTIs: Harris and Olukoga showed that if seatbelt usage increased in urban areas by an additional 16% from a baseline rate of 32%, RTIs could decrease by 9.5% (17). Assuming linearity, this translates to a savings of USD 2.72 million in a single South African province (17). With the exception of this study, cost analyses of RTI prevention interventions are absent in South Africa.

There are many ways to describe the costs of RTIs. However, in our review of the data, we found that the published studies described above often did not define the types of costs that were included. The cost of health care includes much more than the upfront hospital bills. Costs can be categorized into three groups: provider costs defined as the organizing and operating costs of health sector; patient costs defined as the costs borne by the patients and their families; and societal costs or costs borne externally to the health sector and the patient (18). These important cost distinctions are not made in the current South African published literature.

As such, it is difficult to compare the costs between studies and even understand the economic magnitude of RTI in South Africa. Moreover, our review of the South African literature of the RTI cost data available over the last two decades found that, arguably, one of the most important categories of costs-effectiveness – cost analysis – is lacking (19). In view of the dearth of RTI cost-analysis data and to understand the extent to which RTI-related costs in South Africa compare to costs in other LMICs, we reviewed published economic evaluations of RTI-related prevention in LMICs.

## Methods

Six databases, including PubMed/Medline (20), Embase (21), the Cochrane Library (22), EconLit (23), Econbase (24), and the National Health Service Economic Evaluation Database (25), were searched to identify articles containing information on the costs associated with RTIs in LMICs. Searches were not limited by year or language. Citations and reference lists were reviewed to further identify relevant studies (26). Our search terms are provided in Appendix.

All citations were imported into an electronic database (Refworks^®^, Proquest, Bethesda, MD) and two reviewers independently assessed the identified studies. Titles and abstracts were screened for initial exclusion. Articles were excluded if they were not relevant to LMICs and RTIs, and did not discuss economic evaluations. Review articles, commentaries, and editorials were excluded. The full texts of articles were then obtained and reviewed using the same exclusion criteria. Studies were included if they described an economic evaluation of RTIs in a LMIC. Information was extracted using a standardized data form and tabulated in Microsoft Excel^®^ for the following categories: study aim, setting, sample population, type of economic evaluation, methods, data sources, and findings.

As part of a descriptive analysis of the data, studies were grouped according to the type of economic evaluation that best reflected their aim, design, and methods. They included partial and full economic evaluations. Partial evaluations included studies that examined either the costs of the output (RTIs) or input (prevention interventions), but not both (18). For the purposes of this review, these studies were classified as either cost-of-injury or cost-of-prevention studies. Cost-of-injury studies categorized costs as medical costs, costs associated with loss of productivity, and total costs (27, 28). Loss of productivity was attributed to absence from work and premature death (29). Cost-of-prevention studies described the costs associated with purchasing an RTI-related safety device or implementing a prevention intervention.

Full economic evaluation studies, which include cost-effectiveness analyses (CEAs), cost-benefit analyses (CBAs), and cost-utility analyses (CUAs), compare the relative costs and outcomes of two or more interventions. CEAs report costs as a ratio: the denominator is a gain in health, such as a year of life, and the numerator is the cost associated with that health gain. CBAs report costs in terms of willingness to pay (WTP) for injury prevention tools. CUAs, a variant of CEA, report consequences in terms of preference-based measures of health, such as quality-adjusted life years (QALYs) (18).

## Results

**Fig. 3:**
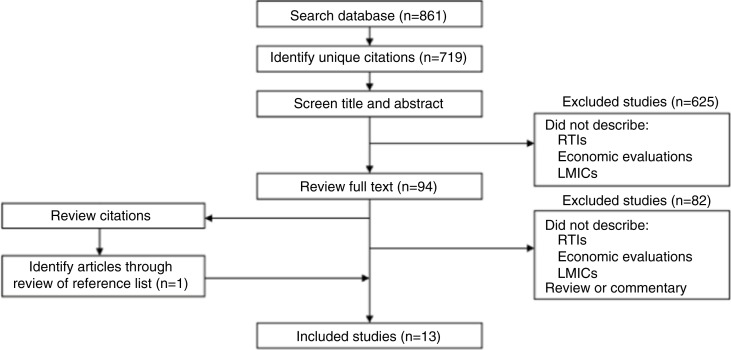
Search strategy flowchart.

Our review identified 13 articles that met inclusion criteria (Fig. 3). In one article, four CEAs were performed using baseline data from four different studies (30). For the purposes of this review, we present these analyses separately, giving a total of 16 economic evaluation studies (Table 2).

**Table 2:** Published studies that describe economic evaluations of road traffic injuries (RTIs) in low- and middle-income countries (costs reported in USD 2010)

Author, Year	Country	WHO region	Intervention/study aim	Study setting	Study sample	Methods and data source	Findings
Cost of injury							
Al-Masaeid, 1998 (31)	Jordan	EMRO	Estimate the cost of RTIs.	National level	15,375 RTIs	Cost of RTIs from police, insurance, and hospital data	Mean RTI cost per injured person: $4,200
Hijar, 2004 (32)	Mexico	AMRO	Analyses the impact of RTIs on demand for hospital emergency services.	4 urban hospitals	233 RTIs	Cost of RTI from patient interviews	Pedestrians had higher health care costs and 80% paid out of pocket, compared to 45% of drivers and passengers
Anh, 2005 (33)	Vietnam	SEARO	Estimate the cost of RTIs.	National Level	26,925 RTIs	Cost of RTI from police, court, and insurance data	Mean RTI cost per injured person: $8,770
Riewpaiboon 2008 (34)	Thailand	SEARO	Estimate the cost of RTIs.	District hospital	200 RTIs	Cost of RTI from hospital records	Mean RTI cost per injured person: $2,980
Riewpaiboon 2008 (35)	Thailand	SEARO	Develop a drug cost model for RTI patients.	Urban hospital	3,723 RTIs	Cost of RTI described in a drug cost model	Mean predicted RTI drug cost per injured person: $21
Li, 2011 (36)	China	WPRO	Estimate the cost of bicycle injuries.	Urban city	550 bicycle-related injuries	Cost of bicycle injuries from hospital records and government data	Mean bicycle-related injury costs per injured person: $4,330. Total productivity loss: $136 million (10.9% GDP)
Parkinson, 2014 (37)	South Africa	AFRO	Estimate the cost of RTIs.	District hospital	100 RTIs	Cost of RTI from hospital records	Mean RTI cost per injured person: $6,610
Cost of RTI prevention							
Bishai, 2003 (38)	Uganda, Pakistan	AFRO, EMRO	Assess the effectiveness of road safety investments.	National level	Model	Analysis of road safety expenditures data	National cost per capita on road safety Pakistan: $0.09; Uganda: $0.12
Hendrie, 2004 (39)	Albania, China, Philippines Thailand, Venezuela, Vietnam	EURO, WPRO, SEARO AMRO	Compare the affordability of safety devices.	Urban settings	Retail stores and internet vendors	Affordability defined as hours needed to work to afford safety device	Mean cost and number of factory hours needed to work to pay for safety devices: Car seat: $102; 30.9 h Booster seat: $98.7; 36.7 h Motorcycle helmet: $15.7; 4.1 h
Cost-benefit analysis							
Pham, 2008 (40)	Vietnam	WPRO	Estimate WTP for motorcycle helmets.	Urban city	414 households	Households’ WTP	A $3.99 government subsidy resulted in a 99% WTP for a motorcycle helmet
Cost-utility analysis							
Tsauo, 1999 (41)	Taiwan	WPRO	Estimate the costs and effectiveness of motorcycle helmet enforcement.	Urban city	99 RTIs with head injury	QAST (42, 43)	Motorcycle helmet enforcement could decrease RTI-related head injuries by 1,300, or 6,240 QALYs gained
Cost-effectiveness analysis							
Bishai, 2006 (30)	China	WPRO	Estimate the costs and effectiveness of bicycle helmet enforcement.	Provincial level	Model	CEA modelling using data from Li, 1997^44^	Bicycle helmet enforcement could decrease RTI-related head injuries by 85% or $131 per DALY averted
Bishai, 2006 (30)	China	WPRO	Estimate the costs and effectiveness of motorcycle helmet enforcement.	National Level	Model	CEA modelling using data from Zhang, 2004 (45) and Ichikawa, 2003 (46)	Motorcycle helmet enforcement could decrease RTI-related head injuries by 41% or $572 per DALY averted
Bishai, 2006 (30)	Brazil	AMRO	Estimate the costs and effectiveness of traffic enforcement.	WHO regions	Model	CEA modelling using data from Poli de Figueiredo, 2001 (47)	Traffic enforcement could decrease RTI-related deaths by 25% or $78.4 per DALY averted
Bishai, 2006 (30)	Ghana	AFRO	Estimate the costs and effectiveness of speed bumps.	National level	Model	CEA modelling using data from Afukaar, 2003 (48)	Speed bumps could decrease RTI-related deaths by 10% or $10.9 per DALY averted
Bishai, 2008 (49)	Uganda	AFRO	Estimate the costs and effectiveness of traffic enforcement.	Urban city	10 police stations	ARIMA and Poisson regression	Traffic enforcement could decrease RTI-related deaths by 17% or $669 per death averted
Chisholm, 2008 (50)	All countries	All regions	Estimate the costs and effectiveness of multiple RTI interventions.	All WHO regions	Model	CEA modelling	DALYs saved range from 415 to 425,093 or $1,380–$5,400 per DALY averted

ARIMA: autoregressive integrated moving average; CEA: cost-effectiveness analysis; DALYs: disability-adjusted life years; QALYs: quality-adjusted life years; QAST: quality-adjusted survival time WTP: willingness to pay.

Of the six cost-of-injury studies, four described the average RTI costs per injured person in terms of total, medical, and loss of productivity costs (Table 3) (31, 33, 34, 36). Total costs ranged between USD 2,980 and USD 8,770. The majority of costs were due to loss of productivity (63–96% of total costs). Medical treatment accounted for 1–14% of total costs. South Africa's cost estimates were crudely two to four times higher than costs reported from Jordan, Thailand, Vietnam, and China. These comparisons should, however, be cautiously considered; each study reported different cost standards and included varying cost components, data sources, study sample populations, dates, and settings. Additionally, these four studies were conducted in four different countries and three different regions, with differing medical care costs and GDPs per capita, further limiting cost comparisons, although the use of international dollars can enhance comparability.

**Table 3:** Estimates of costs of road traffic and bicycle injuries per injured person (costs reported in USD 2010)

Injury	Country	Year data were collected	Injured persons in study (n)	Medical costs	Loss of productivity costs	Total costs
Road traffic injuries	South Africa (12)	1998	80,622	$990	$5,486	$16,200^a^
	Jordan (31)	1996	15,927	$473	$1,630	$4,200^b^
	Thailand (34)	2004	200	$93	$2,860	$2,980^c^
	Vietnam (33)	2004	26,925	$1,260	$3,810	$8,770^d^
	South Africa (37)	2014	100	$6,610	N/A	N/A
Bicycle injuries	China (36)	2004	36,705	$58	$3,760	$4,330^e^

a Total costs include medical, loss of productivity, property damage (including vehicle damage, damage to goods carried, and damage to fixed property), pain and suffering, insurance administrative, legal, policy and promotion, and towing costs.

b Total costs include medical, loss of productivity (output), temporary losses, community and family loses, and pain and suffering.

c Total costs include: medical costs, loss of productivity, property damage (including vehicle damage, damage to goods, and damage to fixed property), pain and suffering, insurance administrative costs, legal costs, and funeral costs.

d Total costs include medical costs and loss of productivity costs.

e Total costs include total medical costs and loss of productivity.

In addition to the cost of injury, our review highlighted studies that described the cost of RTI prevention projects. Two studies described the national costs per capita invested in RTI prevention in Uganda and Pakistan and the mean cost of safety restraints in four WHO regions (38, 39). However, without a complete understanding of the context in which the data were collected, the results must be interpreted cautiously. Only two studies, both from Vietnam, explored this in the context of motorcycle helmets (40, 41). Although these studies present interesting findings regarding the acceptance of motorcycle helmet usage among the study participants, we note that the application and use of these methods in other regions are limited in the published literature.

Table 4 presents findings from CEA models regarding RTI prevention interventions in terms of the cost per disability-adjusted life years (DALYs) averted. Many of these analyses are highlighted in the second edition of *Disease Control Priorities in Developing Countries* (30, 49–51). These interventions include bicycle and motorcycle helmet usage, traffic enforcement, and the construction of speed bumps. Findings suggest that at USD 10.9 per DALY averted, speed bumps may be one of the most cost-effective interventions, followed by seatbelt usage and bicycle helmet enforcement at USD 101 and USD 131 per DALY averted, respectively (17, 30). Traffic enforcement, according to three different models, ranged from USD 78.4 to USD 1,860 per DALY averted (30, 49, 50).

**Table 4:** Annualized costs and DALYs averted of road traffic injury (RTI) prevention interventions

Intervention	Author, year	Study or model location	Cost per DALY averted
Traffic enforcement	Bishai, 2006 (30)	All WHO regions	$78.4
	Bishai, 2008 (49)	Uganda	$96
Speed bumps	Bishai, 2006 (30)	Ghana	$10.9
Speed limit enforcement via mobile cameras	Chisholm, 2008 (50)	AfroE	$77,200
Bicycle helmet enforcement	Bishai, 2006 (30)	China	$131
	Chisholm, 2008 (50)	AfroE	$51,400
Motorcycle helmet enforcement	Bishai, 2006 (30)	China	$572
	Chisholm, 2008 (50)	AfroE	$8,680
Seatbelt usage	Harris, 2005 (17)	South Africa	$28.70
	Chisholm, 2008 (50)	AfroE	$22,400
Drink driving laws and enforcement via breath-testing	Chisholm, 2008 (50)	AfroE	$51,300

## Discussion

### Costing implications for South Africa

In the South African context of a quadruple burden of disease, RTIs place a significant burden on a society and health care system already faced with competing priorities. In addition to the growing burden of injuries, South Africa must contend with the ongoing HIV and tuberculosis epidemics, the exploding burden of obesity-related non-communicable diseases, and an unfinished agenda to address maternal and child mortality (2). Evidence-based studies are needed to show the costs and affordability of effective interventions, particularly how they relate to South Africa's major RTI risk factors: lack of pedestrian safety measures, alcohol misuse, aggressive driving, and limited seatbelt use. Policymakers are more likely to act if they understand the financial implications, especially for budgets already under pressure. Full economic evaluations, such as CEAs, are appropriate tools to achieve this: they describe the health benefits gained, and also the costs saved.

From the literature review, and as outlined in Table 5, the only full economic evaluation conducted in South Africa relates to seatbelt usage. Moreover, the societal costs associated with RTIs were not included in any of the reviewed studies. In South Africa, there is no practical methodology in place to value the household costs of injury-related illness. Due to high unemployment rates, the use of average salaries may not be a good measure, particularly in rural areas where unemployment is highest. Although there are methods that can be used to value these household costs, and it is possible to use more than one method with sensitivity analysis, methodological development is needed to include broader societal costs in economic evaluations of RTIs in South Africa.

**Table 5:** Summary of availability of road traffic injury (RTI) cost-effectiveness studies

	Are cost-effectiveness studies available?	
	
RTI intervention	In LMICs	In South Africa
Traffic enforcement	Yes	No
Speed bumps	Yes	No
Alcohol misuse	Yes	No
Bicycle helmet enforcement	Yes	No
Motorcycle helmet enforcement	Yes	No
Seatbelt usage	Yes	Yes

LMICs: low- and middle-income countries.

We propose that South African surveillance systems already in place to collect demographic RTI data, such as the NIMSS and NDOT, could expand their scope of work to include provider, patient, and societal costing data. This could enhance not only our understanding of the costs associated with RTIs, but also allow policy makers to use such data as evidence to invest in RTI prevention. Recently published economic evaluation guidelines, such as the Consolidated Health Economic Evaluation Reporting Standards, offer methods to conduct and report economic evaluations (52). These resources would allow South Africa to move forward to improve data collection and, ultimately, health resource allocation.

Context-specific evidence for RTI risk factors is critical for informing and implementing targeted interventions. In South Africa, we know that major RTI risk factors are aggressive driving, lack of pedestrian safety measures, limited seatbelt use, and alcohol misuse. As such, some of the ‘best buys’ from other LMICs might be applicable in South Africa. For example, at USD 10.9 per DALY averted, speed bumps may be one of the most cost-effective interventions, followed by seatbelt usage and bicycle helmet enforcement at USD 101 and USD 131 per DALY averted, respectively (17, 30). Economies of scale could also be considered for the roadside enforcement of traffic codes, which may only incur incremental costs for monitoring seatbelt use (53). With regard to drinking-and-driving campaigns, interventions that require breathalyzers might be expensive but effective (50). Weighing the costs of legislating, regulating, and enforcing the regional trade of alcohol against the costs of lost lives and productivity from alcohol-related RTIs could be a comparison to use the point of departure for performing an economic analysis (54, 55).

A key aspect of the Decade of Action for Road Safety 2011–2020 is to support research that will provide data not only in terms of road traffic deaths and injuries but also in terms of costs (56). Thus far, the majority of the evidence focused on the cost-effectiveness of injury prevention has taken place in HICs in which less than 10% of the global burden of traffic injury occurs (57–60).

Strong political will, capacity enhancement, and cultural applicability are fundamental to addressing road injuries. Including many actors, such as business and government, could be transformative. Preventing road crashes will be shaped by factors largely outside the health system, as explicitly acknowledged by the WHO Marmot Commission on Social Determinants of Health (61). The South African National Planning Commission, an expert multi-sector panel, has emphasized RTI prevention as a priority for South Africa by 2030 (62). Context-specific data on the cost-effectiveness of prevention of RTIs in South Africa is essential, but this alone will not prevent injuries.

## Conclusion

Road safety is a growing public health issue in South Africa. Economic evaluations of road safety interventions are needed to understand the cost of RTIs and inform policy makers about choices between competing spending priorities.

## Paper context

Context-specific information on the cost-effectiveness of RTI prevention strategies in South Africa is essential for road safety advocacy. The current published literature describing the costs of RTIs in South Africa do not define the full scope of the costs incurred, and there are no cost-effectiveness analyses on interventions apart from seatbelt use. Surveillance systems should consider introducing data on costs, especially societal costs. This information can be used to better inform choices and trade-offs between competing spending priorities.

## Appendix: Key terms formatted for PubMed

(‘Road traffic injury’[All Fields] OR ‘road traffic injuries’[All Fields]) AND (“cost-benefit analysis”[MeSH Terms] OR ‘cost effectiveness’[All Fields] OR ‘cost analysis’[All Fields]) AND (“Cote d'Ivoire”[All Fields] OR “eritrea”[MeSH Terms] OR (“eritrea”[MeSH Terms] OR “eritrea”[All Fields]) OR “ethiopia”[MeSH Terms] OR (“ethiopia”[MeSH Terms] OR “ethiopia”[All Fields]) OR “gambia”[MeSH Terms] OR (“gambia”[MeSH Terms] OR “gambia”[All Fields]) OR “ghana”[MeSH Terms] OR (“ghana”[MeSH Terms] OR “ghana”[All Fields]) OR “guinea”[MeSH Terms] OR (“guinea”[MeSH Terms] OR “guinea”[All Fields]) OR “guinea-bissau”[MeSH Terms] OR (“guinea-bissau”[MeSH Terms] OR “guinea-bissau”[All Fields] OR (“guinea”[All Fields] AND “bissau”[All Fields]) OR “guinea bissau”[All Fields]) OR “haiti”[MeSH Terms] OR (“haiti”[MeSH Terms] OR “haiti”[All Fields]) OR “india”[MeSH Terms] OR (“india”[MeSH Terms] OR “india”[All Fields]) OR “kenya”[MeSH Terms] OR (“kenya”[MeSH Terms] OR “kenya”[All Fields]) OR Democratic[All Fields] AND (“korea”[All Fields] OR “korea”[MeSH Terms]) OR “Kyrgyz Republic”[All Fields] OR “Lao PDR”[All Fields] OR “lesotho”[MeSH Terms] OR (“lesotho”[MeSH Terms] OR “lesotho”[All Fields]) OR “liberia”[MeSH Terms] OR (“liberia”[MeSH Terms] OR “liberia”[All Fields]) OR “madagascar”[MeSH Terms] OR (“madagascar”[MeSH Terms] OR “madagascar”[All Fields]) OR “malawi”[MeSH Terms] OR (“malawi”[MeSH Terms] OR “malawi”[All Fields]) OR “mali”[MeSH Terms] OR (“mali”[MeSH Terms] OR “mali”[All Fields]) OR “mauritania”[MeSH Terms] OR (“mauritania”[MeSH Terms] OR “mauritania”[All Fields]) OR “moldova”[MeSH Terms] OR (“moldova”[MeSH Terms] OR “moldova”[All Fields]) OR “mongolia”[MeSH Terms] OR (“mongolia”[MeSH Terms] OR “mongolia”[All Fields]) OR “mozambique”[MeSH Terms] OR (“mozambique”[MeSH Terms] OR “mozambique”[All Fields]) OR “myanmar”[MeSH Terms] OR (“myanmar”[MeSH Terms] OR “myanmar”[All Fields]) OR “nepal”[MeSH Terms] OR (“nepal”[MeSH Terms] OR “nepal”[All Fields]) OR “nicaragua”[MeSH Terms] OR (“nicaragua”[MeSH Terms] OR “nicaragua”[All Fields]) OR “niger”[MeSH Terms] OR (“niger”[MeSH Terms] OR “niger”[All Fields]) OR “nigeria”[MeSH Terms] OR (“nigeria”[MeSH Terms] OR “nigeria”[All Fields]) OR “North Korea”[All Fields] OR “DPRK”[All Fields] OR “pakistan”[MeSH Terms] OR (“pakistan”[MeSH Terms] OR “pakistan”[All Fields]) OR “Papua New Guinea”[All Fields] OR “rwanda”[MeSH Terms] OR (“rwanda”[MeSH Terms] OR “rwanda”[All Fields]) OR “Sao Tome and Principe”[All Fields] OR “senegal”[MeSH Terms] OR (“senegal”[MeSH Terms] OR “senegal”[All Fields]) OR “Sierra Leone”[All Fields] OR “Solomon Islands”[All Fields] OR “somalia”[MeSH Terms] OR (“somalia”[MeSH Terms] OR “somalia”[All Fields]) OR “sudan”[MeSH Terms] OR (“sudan”[MeSH Terms] OR “sudan”[All Fields]) OR “tajikistan”[MeSH Terms] OR (“tajikistan”[MeSH Terms] OR “tajikistan”[All Fields]) OR “tanzania”[MeSH Terms] OR (“tanzania”[MeSH Terms] OR “tanzania”[All Fields]) OR “east timor”[All Fields] OR “east timor”[MeSH Terms] OR (“east timor”[MeSH Terms] OR (“east”[All Fields] AND “timor”[All Fields]) OR “east timor”[All Fields] OR (“timor”[All Fields] AND “leste”[All Fields]) OR “timor leste”[All Fields]) OR “togo”[MeSH Terms] OR (“togo”[MeSH Terms] OR “togo”[All Fields]) OR “uganda”[MeSH Terms] OR (“uganda”[MeSH Terms] OR “uganda”[All Fields]) OR “uzbekistan”[MeSH Terms] OR (“uzbekistan”[MeSH Terms] OR “uzbekistan”[All Fields]) OR “vietnam”[MeSH Terms] OR (“vietnam”[MeSH Terms] OR “vietnam”[All Fields]) OR “yemen”[MeSH Terms] OR (“yemen”[MeSH Terms] OR “yemen”[All Fields]) OR “Republic of Yemen”[All Fields] OR “democratic republic of the congo”[All Fields] OR “democratic republic of the congo”[MeSH Terms] OR (“democratic republic of the congo”[MeSH Terms] OR (“democratic”[All Fields] AND “republic”[All Fields] AND “congo”[All Fields]) OR “democratic republic of the congo”[All Fields] OR “zaire”[All Fields]) OR “zambia”[MeSH Terms] OR (“zambia”[MeSH Terms] OR “zambia”[All Fields]) OR “zimbabwe”[MeSH Terms] OR (“zimbabwe”[MeSH Terms] OR “zimbabwe”[All Fields]) OR “albania”[MeSH Terms] OR (“albania”[MeSH Terms] OR “albania”[All Fields]) OR “algeria”[MeSH Terms] OR (“algeria”[MeSH Terms] OR “algeria”[All Fields]) OR “angola”[MeSH Terms] OR (“angola”[MeSH Terms] OR “angola”[All Fields]) OR “armenia”[MeSH Terms] OR (“armenia”[MeSH Terms] OR “armenia”[All Fields]) OR “azerbaijan”[MeSH Terms] OR (“azerbaijan”[MeSH Terms] OR “azerbaijan”[All Fields]) OR “byelarus”[MeSH Terms] OR (“byelarus”[MeSH Terms] OR “byelarus”[All Fields] OR “belarus”[All Fields]) OR “bolivia”[MeSH Terms] OR (“bolivia”[MeSH Terms] OR “bolivia”[All Fields]) OR “Bosnia and Herzegovina”[All Fields] OR “brazil”[MeSH Terms] OR (“brazil”[MeSH Terms] OR “brazil”[All Fields]) OR “bulgaria”[MeSH Terms] OR (“bulgaria”[MeSH Terms] OR “bulgaria”[All Fields]) OR “Cape Verde”[All Fields] OR “china”[MeSH Terms] OR (“china”[MeSH Terms] OR “china”[All Fields]) OR “colombia”[MeSH Terms] OR (“colombia”[MeSH Terms] OR “colombia”[All Fields]) OR “cuba”[MeSH Terms] OR (“cuba”[MeSH Terms] OR “cuba”[All Fields]) OR “djibouti”[MeSH Terms] OR (“djibouti”[MeSH Terms] OR “djibouti”[All Fields]) OR “Dominican Republic”[All Fields] OR “ecuador”[MeSH Terms] OR (“ecuador”[MeSH Terms] OR “ecuador”[All Fields]) OR “egypt”[MeSH Terms] OR (“egypt”[MeSH Terms] OR “egypt”[All Fields]) OR “Arab Republic of Egypt”[All Fields] OR “El Salvador”[All Fields] OR “fiji”[MeSH Terms] OR (“fiji”[MeSH Terms] OR “fiji”[All Fields]) OR “georgia republic”[MeSH Terms] OR “georgia”[tiab] OR “georgia (republic)”[MeSH Terms] OR “guatemala”[MeSH Terms] OR (“guatemala”[MeSH Terms] OR “guatemala”[All Fields]) OR “guyana”[MeSH Terms] OR (“guyana”[MeSH Terms] OR “guyana”[All Fields]) OR “honduras”[MeSH Terms] OR (“honduras”[MeSH Terms] OR “honduras”[All Fields]) OR “indonesia”[MeSH Terms] OR (“indonesia”[MeSH Terms] OR “indonesia”[All Fields]) OR “iran”[MeSH Terms] OR (“iran”[MeSH Terms] OR “iran”[All Fields]) OR “Islamic Republic of Iran”[All Fields] OR “iraq”[MeSH Terms] OR (“iraq”[MeSH Terms] OR “iraq”[All Fields]) OR “jamaica”[MeSH Terms] OR (“jamaica”[MeSH Terms] OR “jamaica”[All Fields]) OR “jordan”[MeSH Terms] OR (“jordan”[MeSH Terms] OR “jordan”[All Fields]) OR “kazakhstan”[MeSH Terms] OR (“kazakhstan”[MeSH Terms] OR “kazakhstan”[All Fields]) OR “micronesia”[All Fields] OR “micronesia”[MeSH Terms] OR (“micronesia”[MeSH Terms] OR “micronesia”[All Fields] OR “kiribati”[All Fields]) OR “macedonia republic”[MeSH Terms] OR (“macedonia (republic)”[MeSH Terms] OR (“macedonia”[All Fields] AND “(republic)”[All Fields]) OR “macedonia (republic)”[All Fields] OR “macedonia”[All Fields]) OR “FYR of Macedonia”[All Fields] OR “Former Yugoslav Republic of Macedonia”[All Fields] OR “indian ocean islands”[All Fields] OR “indian ocean islands”[MeSH Terms] OR (“indian ocean islands”[MeSH Terms] OR (“indian”[All Fields] AND “ocean”[All Fields] AND “islands”[All Fields]) OR “indian ocean islands”[All Fields] OR “maldives”[All Fields]) OR “Marshall Islands”[All Fields] OR “micronesia”[MeSH Terms] OR (“micronesia”[MeSH Terms] OR “micronesia”[All Fields]) OR “Federated States of Micronesia”[All Fields] OR “morocco”[MeSH Terms] OR (“morocco”[MeSH Terms] OR “morocco”[All Fields]) OR “namibia”[MeSH Terms] OR (“namibia”[MeSH Terms] OR “namibia”[All Fields]) OR “paraguay”[MeSH Terms] OR (“paraguay”[MeSH Terms] OR “paraguay”[All Fields]) OR “peru”[MeSH Terms] OR (“peru”[MeSH Terms] OR “peru”[All Fields]) OR “philippines”[MeSH Terms] OR (“philippines”[MeSH Terms] OR “philippines”[All Fields]) OR “romania”[MeSH Terms] OR (“romania”[MeSH Terms] OR “romania”[All Fields]) OR “samoa”[MeSH Terms] OR (“samoa”[MeSH Terms] OR “samoa”[All Fields]) OR “Serbia and Montenegro”[All Fields] OR “Sri Lanka”[All Fields] OR “suriname”[MeSH Terms] OR (“suriname”[MeSH Terms] OR “suriname”[All Fields]) OR “swaziland”[MeSH Terms] OR (“swaziland”[MeSH Terms] OR “swaziland”[All Fields]) OR “Syrian Arab Republic”[All Fields] OR “syria”[MeSH Terms] OR (“syria”[MeSH Terms] OR “syria”[All Fields]) OR “thailand”[MeSH Terms] OR (“thailand”[MeSH Terms] OR “thailand”[All Fields]) OR “tonga”[MeSH Terms] OR (“tonga”[MeSH Terms] OR “tonga”[All Fields]) OR “tunisia”[MeSH Terms] OR (“tunisia”[MeSH Terms] OR “tunisia”[All Fields]) OR “turkmenistan”[MeSH Terms] OR (“turkmenistan”[MeSH Terms] OR “turkmenistan”[All Fields]) OR “ukraine”[MeSH Terms] OR (“ukraine”[MeSH Terms] OR “ukraine”[All Fields]) OR “vanuatu”[MeSH Terms] OR (“vanuatu”[MeSH Terms] OR “vanuatu”[All Fields]) OR “West Bank and Gaza”[All Fields] OR “American Samoa”[All Fields] OR “Antigua and Barbuda”[All Fields] OR “argentina”[MeSH Terms] OR (“argentina”[MeSH Terms] OR “argentina”[All Fields]) OR “barbados”[MeSH Terms] OR (“barbados”[MeSH Terms] OR “barbados”[All Fields]) OR “belize”[MeSH Terms] OR (“belize”[MeSH Terms] OR “belize”[All Fields]) OR “botswana”[MeSH Terms] OR (“botswana”[MeSH Terms] OR “botswana”[All Fields]) OR “chile”[MeSH Terms] OR (“chile”[MeSH Terms] OR “chile”[All Fields]) OR “Costa Rica”[All Fields] OR “croatia”[MeSH Terms] OR (“croatia”[MeSH Terms] OR “croatia”[All Fields]) OR “Czech Republic”[All Fields] OR “dominica”[MeSH Terms] OR (“dominica”[MeSH Terms] OR “dominica”[All Fields]) OR “Equatorial Guinea”[All Fields] OR “estonia”[MeSH Terms] OR (“estonia”[MeSH Terms] OR “estonia”[All Fields]) OR “gabon”[MeSH Terms] OR (“gabon”[MeSH Terms] OR “gabon”[All Fields]) OR “grenada”[MeSH Terms] OR (“grenada”[MeSH Terms] OR “grenada”[All Fields]) OR “hungary”[MeSH Terms] OR (“hungary”[MeSH Terms] OR “hungary”[All Fields]) OR “latvia”[MeSH Terms] OR (“latvia”[MeSH Terms] OR “latvia”[All Fields]) OR “lebanon”[MeSH Terms] OR (“lebanon”[MeSH Terms] OR “lebanon”[All Fields]) OR “libya”[MeSH Terms] OR (“libya”[MeSH Terms] OR “libya”[All Fields]) OR “lithuania”[MeSH Terms] OR (“lithuania”[MeSH Terms] OR “lithuania”[All Fields]) OR “malaysia”[MeSH Terms] OR (“malaysia”[MeSH Terms] OR “malaysia”[All Fields]) OR “mauritius”[MeSH Terms] OR (“mauritius”[MeSH Terms] OR “mauritius”[All Fields]) OR “comoros”[All Fields] OR “comoros”[MeSH Terms] OR (“comoros”[MeSH Terms] OR “comoros”[All Fields] OR “mayotte”[All Fields]) OR “mexico”[MeSH Terms] OR (“mexico”[MeSH Terms] OR “mexico”[All Fields]) OR “Northern Mariana Islands”[All Fields] OR “oman”[MeSH Terms] OR (“oman”[MeSH Terms] OR “oman”[All Fields]) OR “palau”[MeSH Terms] OR (“palau”[MeSH Terms] OR “palau”[All Fields]) OR “panama”[MeSH Terms] OR (“panama”[MeSH Terms] OR “panama”[All Fields]) OR “poland”[MeSH Terms] OR (“poland”[MeSH Terms] OR “poland”[All Fields]) OR “Russian Federation”[All Fields] OR “seychelles”[MeSH Terms] OR (“seychelles”[MeSH Terms] OR “seychelles”[All Fields]) OR “Slovak Republic”[All Fields] OR “South Africa”[All Fields] OR “St. Kitts and Nevis”[All Fields] OR “St. Lucia”[All Fields] OR “St. Vincent and the Grenadines”[All Fields] OR “Trinidad and Tobago”[All Fields] OR “turkey”[MeSH Terms] OR (“turkey”[MeSH Terms] OR “turkey”[All Fields]) OR “uruguay”[MeSH Terms] OR (“uruguay”[MeSH Terms] OR “uruguay”[All Fields]) OR “venezuela”[MeSH Terms] OR (“venezuela”[MeSH Terms] OR “venezuela”[All Fields]) OR “developing countries”[All Fields] OR “less developed countries”[All Fields] OR “third-world countries”[All Fields] OR “under-developed countries”[All Fields] OR “poOR countries”[All Fields] OR “less developed countries”[All Fields] OR “under developed countries”[All Fields] OR “less developed nations”[All Fields] OR “third world nations”[All Fields] OR “under developed nations”[All Fields] OR “developing nations”[All Fields] OR “poOR nations”[All Fields] OR “poor economies”[All Fields] OR “developing economies”[All Fields] OR “less developed economies”[All Fields] OR “myanmar”[MeSH Terms] OR (“myanmar”[MeSH Terms] OR “myanmar”[All Fields] OR “burma”[All Fields]) OR “Czechoslovakia”[All Fields] OR “Democratic Republic of Congo”[All Fields] OR “French Guiana”[All Fields] OR “East Timor”[All Fields] OR “laos”[MeSH Terms] OR (“laos”[MeSH Terms] OR “laos”[All Fields]) OR “North Korea”[All Fields] OR “Ivory Coast”[All Fields] OR “Republic of Georgia”[All Fields] OR “Republic of Yemen”[All Fields] OR “Republic of Zaire”[All Fields] OR “slovakia”[MeSH Terms] OR (“slovakia”[MeSH Terms] OR “slovakia”[All Fields]) OR “Soviet Union”[All Fields] OR “suriname”[MeSH Terms] OR (“suriname”[MeSH Terms] OR “suriname”[All Fields] OR “surinam”[All Fields]) OR “ussr”[MeSH Terms] OR (“ussr”[MeSH Terms] OR “ussr”[All Fields]) OR “samoa”[MeSH Terms] OR (“samoa”[MeSH Terms] OR “samoa”[All Fields]) OR “yugoslavia”[MeSH Terms] OR (“yugoslavia”[MeSH Terms] OR “yugoslavia”[All Fields]) OR “democratic republic of the congo”[MeSH Terms] OR (“democratic republic of the congo”[MeSH Terms] OR (“democratic”[All Fields] AND “republic”[All Fields] AND “congo”[All Fields]) OR “democratic republic of the congo”[All Fields] OR “zaire”[All Fields]) OR “asia”[MeSH Terms] OR (“asia”[MeSH Terms] OR “asia”[All Fields]) OR “West Indies”[All Fields] OR “polynesia”[MeSH Terms] OR (“polynesia”[MeSH Terms] OR “polynesia”[All Fields]) OR “micronesia”[MeSH Terms] OR (“micronesia”[MeSH Terms] OR “micronesia”[All Fields]) OR “Middle East”[All Fields] OR “africa”[MeSH Terms] OR (“africa”[MeSH Terms] OR “africa”[All Fields]) OR “Latin America”[All Fields] OR “Central America”[All Fields] OR “South America”[All Fields] OR (“west indies”[MeSH Terms] OR (“west”[All Fields] AND “indies”[All Fields]) OR “west indies”[All Fields]) OR “west indies”[MeSH Terms] OR “caribbean region”[MeSH Terms] OR (“west indies”[MeSH Terms] OR (“west”[All Fields] AND “indies”[All Fields]) OR “west indies”[All Fields] OR “caribbean”[All Fields] OR “caribbean region”[MeSH Terms] OR (“caribbean”[All Fields] AND “region”[All Fields]) OR “caribbean region”[All Fields]) OR “caribbean region”[MeSH Terms] OR Hispanola[All Fields] OR “Southeast Asia”[All Fields] OR “Sub-Saharan Africa”[All Fields] OR “Eastern Europe”[All Fields] OR Balkans[All Fields]) NOT (“new mexico”[MeSH Terms]) OR “new mexico”[All Fields] “Developing Countries”[MeSH Terms] OR “Developing Country” [All Fields] OR “Developing Countries” [All Fields] OR “Low Resource Setting” [All Fields] OR “Low Resource Settings” [All Fields])
